# The Prevalence of Underlying Diseases and Comorbidities in COVID-19 Patients; an Updated Systematic Review and Meta-analysis

**Published:** 2020-09-12

**Authors:** Sorour Khateri, Hedyeh Mohammadi, Rozhin Khateri, Yousef Moradi

**Affiliations:** 1 School of Medicine, Kurdistan University of Medical Sciences, Sanandaj, Iran.; 2 Faculty of Nursing and Midwifery, Kurdistan University of Medical Sciences, Sanandaj, Iran.; 3 Social Determinants of Health Research Center, Research Institute for Health Development, Kurdistan University of Medical Sciences, Sanandaj, Iran.

**Keywords:** Comorbidity, Prognosis, COVID-19, Systematic Review, Meta-analysis

## Abstract

**Introduction:**

Gaining knowledge about underlying diseases and associated comorbidities in patients with COVID-19 can be beneficial in developing a proper understanding of the disease prognosis as well as comprehensive management, and treatment of the disease. The aim of this study was to determine the prevalence of underlying diseases and associated comorbidities in COVID-19 patients using a systematic review and meta-analysis.

**Methods:**

Major biomedical electronic databases, including Scopus, PubMed, Web of Science, CINAHL and EMBASE were searched for all relevant literature published in English from January to July 2020. Cross-sectional and retrospective studies reporting the prevalence of comorbid conditions such as acute cardiac injury, acute myocardial infarction, acute kidney injury, acute liver injury, shock, acute respiratory disease, and acute respiratory distress syndrome in patients with COVID-19 were included in the study. After selecting eligible studies, two authors extracted data of each study, independently, and any inconsistency was resolved through discussion with the third reviewer until reaching a consensus. The risk of bias was assessed by two independent research experts using the Newcastle-Ottawa Scale (NOS). The variance in the meta-analyses on prevalence was stabilized by double arcsine transformations.

**Results:**

The pooled prevalence of acute respiratory injury in patients with COVID-19 was estimated as 34% (95% Cl: 10 – 57%). Also, the prevalence of acute kidney injury, acute liver injury, acute respiratory distress syndrome, and shock were estimated as 10% (95% Cl: 6 - 14%), 19% (95% Cl: 10 - 27%), 23 % (95% Cl: 19 - 27%), and 12 % (95% Cl: 5 – 19 %).

**Conclusion:**

According to this meta-analysis, comorbidities such as hypertension, acute liver and kidney injury, acute respiratory distress syndrome, shock, diabetes, and coronary heart disease seem to be a predisposing factor for symptomatic and severe COVID-19 infection.

## Introduction

In late December 2019, a series of unexplained cases of pneumonia were reported in Wuhan, China. The government and health researchers in China took swift steps to control the spread of the epidemic and launched etiological studies. On January 12, 2020, the WHO declared the novel coronavirus (2019-nCoV) epidemic as a Public Health Emergency of International Concern (PHEIC). On February 11, 2020, WHO officially named the disease caused by 2019-nCoV as COVID-19 (1, 2). The novel 2019 Coronavirus (SARS-CoV-2) belongs to the beta-coronavirus group (3). The 2019-nCoV is thought to be transmitted through droplets, close contact, aerosols, and possibly through fecal-oral transmission, and patients in the incubation period can transmit the virus to others (4, 5). The distribution of viral receptor can explain the pathogenic mechanisms, clinical manifestations, and transmission routes of the 2019-nCoV. Angiotensin-converting enzyme 2 (ACE2) has been introduced as the receptor for the 2019-nCoV, which is essential for its entry. Expression of ACE2 in various cells, such as pulmonary AT2 cells, the upper esophagus, epithelial cells, and absorption enterocytes of the ileum and large intestine, may play a role in multi-tissue infection of 2019-nCoV (6, 7). The disease usually causes viral pneumonia similar to influenza often about a week after the onset of the first symptoms, and causes shortness of breath, decreased oxygen saturation in the blood, and death in people with underlying disorders (8). Renal disorders and septic shock have also been identified as other causes of death from COVID-19 (9, 10). Due to the novelty of the disease and the occurrence of most cases in China, the number of deaths or disabilities caused by it is still unknown. The rate of need for intensive care in hospitalized patients in China was reported to be between 23% and 32%, and the mortality rate was reported to be from 4.3% to 15% in the first articles published by Chinese centers (3). With the spread of the disease to 114 countries, COVID-19 outbreak was announced to be a pandemic on March 11, 2020 (2). In other countries of the world, as well as an increase in the number of diagnosed cases with less severe symptoms, led to changes in mortality rates and in the variables affecting death (11). Finding the disease’s accurate mortality rate requires designing targeted cohort studies to more accurately record the number of those affected and patients who die and more consistently identify low-symptom patients (12). Since many hospitalized patients, especially those who are eventually hospitalized in the ICU or die, suffer from comorbidities such as diabetes, hypertension, chronic cardiovascular disease, etc., determining the frequency or prevalence of these underlying diseases and associated comorbidities can be beneficial in developing a proper understanding of the disease prognosis as well as comprehensive management, and treatment of the disease. To find a reliable answer, we performed a systematic review and meta-analysis, which estimated the pooled prevalence of underlying diseases and comorbidities in all patients. These findings may aid in patient management, mortality prevention, and development of policies regarding response to COVID-19 and predicting its outcome. The aim of this study was to determine the prevalence of underlying diseases and associated comorbidities in COVID-19 patients using a systematic review and meta-analysis.

## Methods

We performed this systematic review using the Meta-Analyses of Observational Studies in Epidemiology (MOOSE)(13) and Preferred Reporting Items for Systematic Reviews and Meta-Analyses (PRISMA)(14). 

**Search Terms and Search Strategy**

A comprehensive systematic search was implemented combining text-word and subject heading (MeSH or equivalent) of the following online databases: PubMed (including Medline), Web of Science, Scopus, CINAHL and Embase; searching for articles published from January to July 2020. To search in the electronic databases, we applied all possible keywords related to “COVID-19”, “Coronavirus”, “Acute Cardiac Injury”, “Acute Myocardial Infarction”, “Acute Kidney Injury”, “Acute Liver Injury”, “Shock”, “Acute Respiratory Disease” and “Acute Respiratory Distress Syndrome”. The search strategies in Embase and PubMed are shown in Table 1. The primary search results were received and some of the articles were omitted based on their titles and abstracts. Direct contact with authors was attempted in case there was incomplete information or any clariﬁcation was required. An identical search strategy was conducted in other databases. Further, hand-searching of the key journals and the reference lists of the included papers was also performed. 

**Selection and Screening**

**Figure 1:**
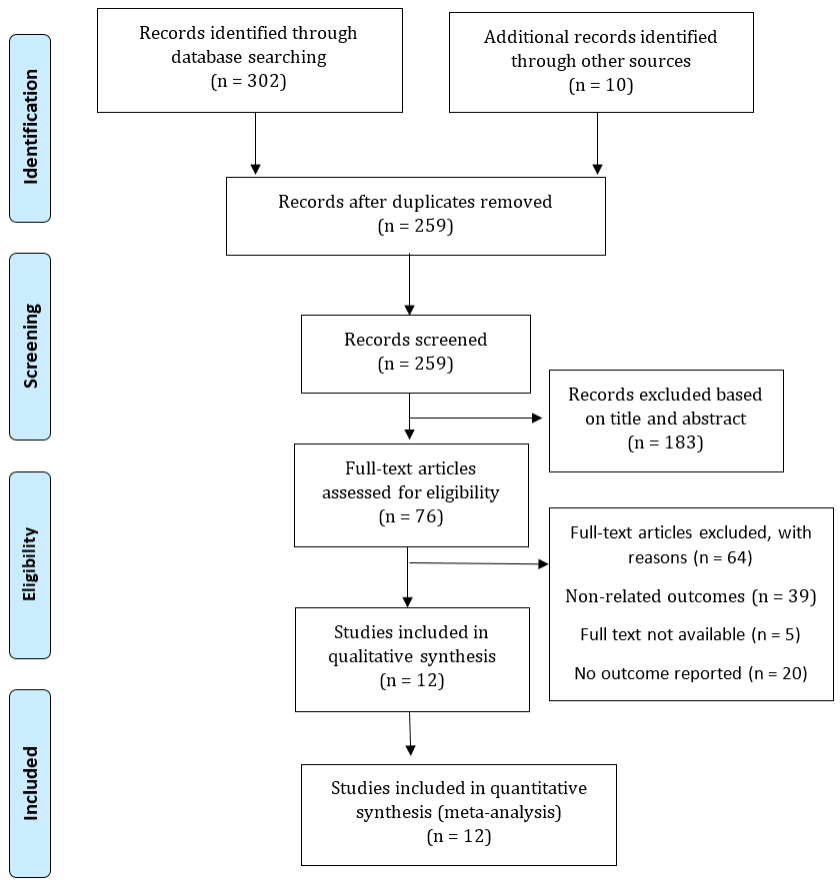
The diagram of study selection

The articles were selected in two steps. First, two independent authors (SKH and RKH) of this study reviewed the articles found, and evaluated them for meeting inclusion and exclusion criteria based on their title and abstract, and then abolished irrelevant studies. Second, the full-text of the remaining articles from the previous stage was extracted and explored independently by each of the authors to determine the eligibility of the articles. Finally, we selected 11 scientific articles about prevalence of comorbidities in patients with COVID-19 (Figure 1). PRISMA diagram was drawn to illustrate the study selection process. To identify any missing studies, we checked the reference list of each selected paper.

**Inclusion criteria**

In this study, full-text articles published as original research in scientific journals were selected in the first step. In addition, studies reporting the prevalence of comorbid conditions like acute cardiac injury, acute myocardial infarction, acute kidney injury, acute liver injury, shock, acute respiratory disease, and acute respiratory distress syndrome in patients with COVID-19 were included in the study. In addition, cross-sectional and retrospective studies published in English, which assessed and reported the number of patients with COVID-19 were included.

**Exclusion criteria**

Reports, brief reports, books, textbooks, dissertations, reviews, letter to the editor, case reports, case studies, landscape articles, symposia, posters, brief communications, unofficial interpretations, non-English articles, and articles unrelated to prevalence of comorbid conditions in patients with COVID-19 were excluded. In addition, studies whose content was not related to the subject of research, or had either incorrect or vague information were excluded.

**Data extraction**

At this stage of the review, an initial data extraction form was prepared. The elements of information were extracted from each article in two parts: general items (first author, publication year, country, age, gender, and study population) and specific items (type of underlying disease, and comorbidity). Then, two authors (SKH and HM) separately reviewed and collected the data for each item. In addition, disagreement between the two authors, if any, was resolved through intervention of a third party. In the next step, the results were analyzed in a descriptive manner and the topics were grouped and meta-analyzed. These items are described in the results section of this article. 

**Risk of Bias**

Qualitative evaluation of studies, based on the Newcastle-Ottawa Quality Assessment Scale (NOS) (15), was performed by two of the authors (YM and RKH). This scale is designed for qualitative evaluation of observational studies. NOS examines each study for six items in three groups; selection, comparability, and exposure. Points are given to each item and the maximum score is 9. Finally, the articles were categorized as low, moderate, and high risk. The Strengthening the Reporting of Observational studies in Epidemiology (STROBE) checklist was also completed for all articles (16, 17).

**Statistical Analysis**

The variance in meta-analyses on prevalence was stabilized by double arcsine transformations. Forest plots, χ2 test (at a significance level of 10%) and I2 index were used to study the heterogeneity among the selected articles. A random-effects model was applied for articles with high heterogeneity (I2>50%); for other cases, a fixed effects model was used. The year of publication and the age of patients were regarded to select a meta-regression considering the source of heterogeneity. Statistical analyses were performed using STATA 14.0 (Stata Corp, College Station, TX, USA) and statistical significance was set at p < 0.05.

## Results

**Study Characteristics**

312 articles were initially retrieved by applying the search strategies in the online databases. Among these articles, 53 duplicate publications were identified and removed. The remaining ones were screened based on their titles and abstracts. 12 articles were selected as the final papers to be analyzed (18-29) (Figure 1). A total of 2393 patients with COVID-19 (1250 Male and 1089 Female) were evaluated through 6 retrospectives, 4 cross-sectional, and 2 cohort studies. Comorbidities assessed in these studies included coronary heart disease, diabetes, hypertension, chronic obstructive pulmonary disease (COPD), acute cardiac injury, acute kidney injury, acute liver injury, acute respiratory distress syndrome and acute respiratory disease. The smallest and largest groups consisted of 41 and 788 patients, respectively. The studies were done in China. Some other diseases such as dementia, cancers, mental disorders, hepatitis B virus, and psychological diseases had also been evaluated by some scientists, which were excluded due to their very low prevalence in our assay (Table 2).

**Quantitative Analysis**

***The pooled prevalence of acute respiratory injury***

**Figure 2:**
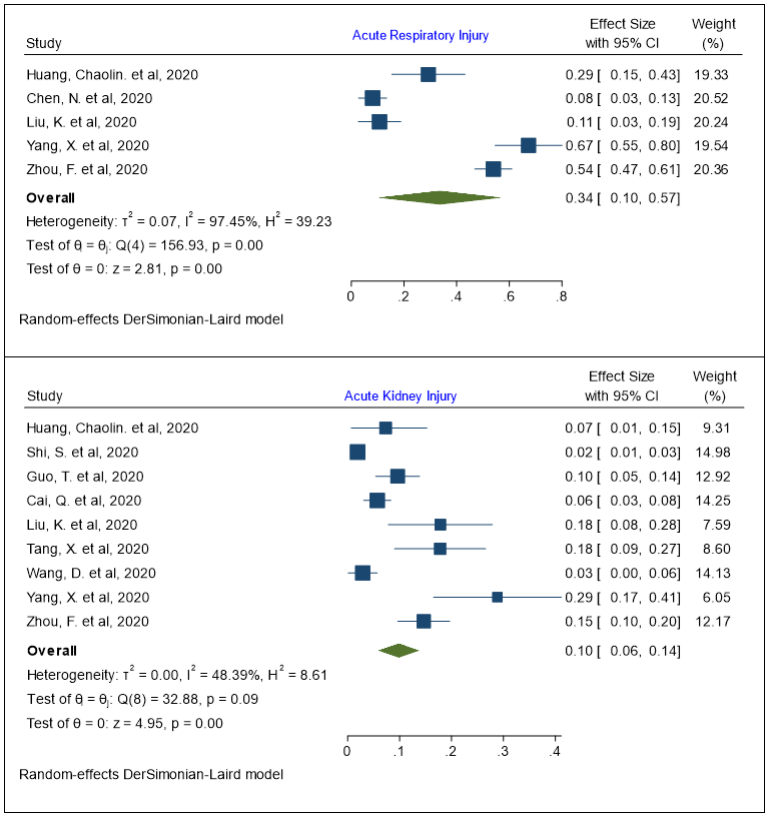
The pooled prevalence of acute respiratory and kidney injury in patients with COVID-19

The lowest reported prevalence rate for acute respiratory injury in patients with COVID-19 was 8% (95% Cl: 3 - 13%) in the study by Chen, N. et al. (21) and the highest acute respiratory injury prevalence rate was 67% (95% Cl: 55- 80%), reported in the retrospective study by Yang, X. et al.(29) In total, the pooled prevalence of acute respiratory injury has been estimated as 34% (95% Cl: 10 - 57%; I^2^= 97.45%; Q test = 156.93; P = 0.001) (Figure 2). Meta regression was used to explore the relationship of the independent variable (age) with the pooled prevalence of acute respiratory injury. The results of meta regression showed that the prevalence of acute respiratory injury has no relationship with age (coefficient: 0.017, P: 0.579, 95% CI: -0.043, 0.078). The pooled prevalence of acute respiratory injury in patients aged 60 years or less was 19% (95% Cl: 1 - 37%; I^2^= 98.58%; Q test = 140.77; P = 0.001) and in patients older than 60 years the prevalence was 43% (95% Cl: 6 - 80%; I^2^= 80.37%; Q test = 5.09; P = 0.02) (Table 3). 

***The pooled prevalence of acute kidney injury***

 The pooled prevalence of acute kidney injury has been estimated as 10% (95% Cl: 6 - 14%; I^2^= 48.39%; Q test = 32.88; P = 0.09) (Figure 2). The lowest and highest reported prevalence rates for acute kidney injury in patients with COVID-19 were 2% (95% Cl: 1 - 3%) and 29% (95% Cl: 17 - 41%) in the studies by Shi, S. et al. (18) and Yang, X. et al.(29), respectively (Figure 2). The results of meta regression showed that the prevalence of acute kidney injury has no relationship with age (coefficient: 0.016, P: 0.648, 95% CI: -0.005, 0.008). The pooled prevalence of acute kidney injury in patients aged 60 years or less was 9 % (95% Cl: 3 - 15%; I^2^= 62.35%; Q test = 5.77; P = 0.07) and in patients older than 60 years the prevalence was 11 % (95% Cl: 5 - 16%; I^2^= 59.32%; Q test = 5.09; P = 0.24) (Table 3). 

***The pooled prevalence of acute liver injury***

**Figure 3:**
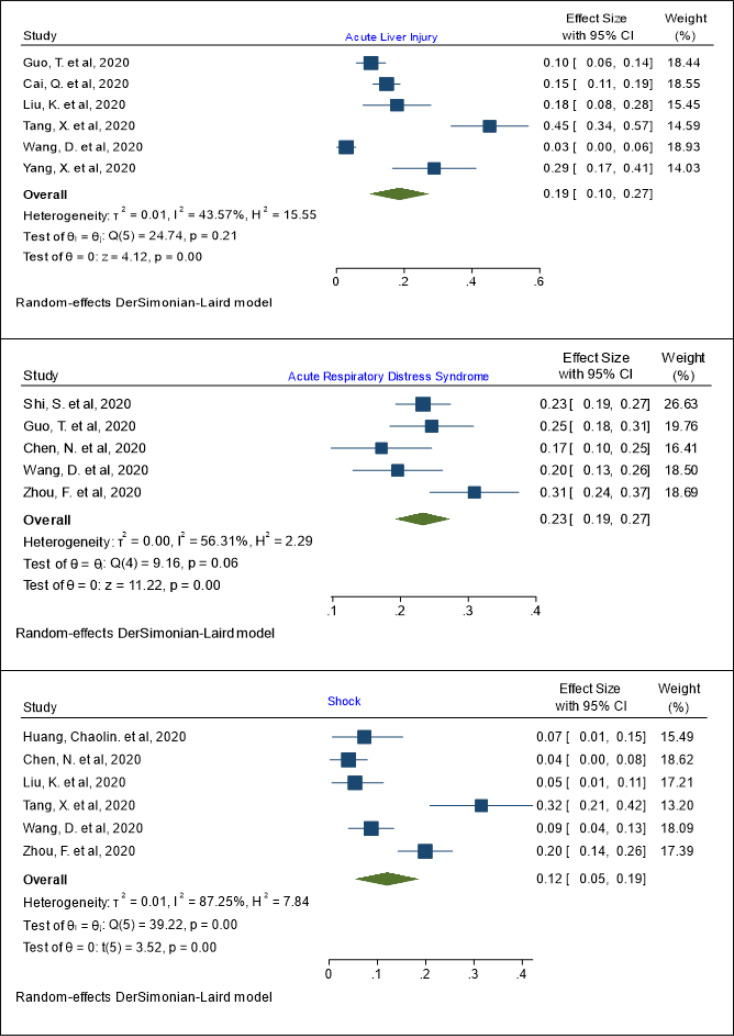
The pooled prevalence of acute liver injury, respiratory distress syndrome, and shock in patients with COVID-19

 The pooled prevalence of acute liver injury has been estimated as 19% (95% Cl: 10 - 27%; I^2^= 43.57%; Q test = 24.47; P = 0.21) (Figure 3). The lowest and highest prevalence rates reported for acute kidney injury in patients with COVID-19 were 3% (95% Cl: 0 - 6%) and 45% (95% Cl: 34 - 57%) in the studies by Wang, D. et al. (26) and Tang, X. et al. (25), respectively (Figure 3). The results of meta regression showed that the prevalence of acute liver injury has no relationship with age (coefficient: 0.012, P: 0.110, 95% CI: -0.002, 0.028). The pooled prevalence of acute kidney injury in patients aged 60 years or less was 15% (95% Cl: 11 - 19%; I^2^= 0.0%; Q test = 0.58; P = 0.31) and in patients older than 60 years the prevalence was 20% (95% Cl: 7 - 34%; I^2^= 55.40%; Q test = 4.99; P = 0.09) (Table 3). 

***The pooled prevalence of acute respiratory distress syndrome***

The lowest prevalence rate reported for acute respiratory distress syndrome in patients with COVID-19 was 17% (95% Cl: 10 - 25%) found in the study by Chen, N. et al. (21) and the highest rate of acute respiratory distress syndrome prevalence was 31% (95% Cl: 24 - 37%), reported in the retrospective study by Zhou, F. et al. (27) In total, the pooled prevalence of acute respiratory distress syndrome has been estimated as 23% (95% Cl: 19 - 27%; I^2^= 56.31%; Q test = 9.16; P = 0.06) (Figure 3). Meta regression was used to explore the relationship of the independent variable (age) with the pooled prevalence of acute respiratory distress syndrome. The results of meta regression showed that the prevalence of acute respiratory distress syndrome has no relationship with age (coefficient: -0.003, P: 0.653, 95% CI: -0.019, 0.012). The pooled prevalence of acute respiratory distress syndrome in patients aged 60 years or less was 25% (95% Cl: 19 - 31%; I^2^= 64.97 %; Q test = 5.71; P = 0.08) and in patients older than 60 years the prevalence was 21% (95% Cl: 15 - 27%; I^2^= 50.58%; Q test = 2.02; P = 0.15) (Table 2). 

***The pooled prevalence of shock***

 The pooled prevalence of shock has been estimated as 12% (95% Cl: 5 – 19 %; I^2^= 87.25%; Q test = 39.22; P = 0.001) (Figure 3). The lowest and highest reported prevalence rates of shock in patients with COVID-19 were 4% (95% Cl: 0 - 8%) and 32% (95% Cl: 21 - 42%) reported in the studies by Chen, N. et al.(21) and Tang, X. et al. (25), respectively (Figure 3). The results of meta regression showed that the prevalence of shock has no relationship with age (coefficient: 0.010, P: 0.094, 95% CI: -0.001, 0.023). The pooled prevalence of shock in patients aged 60 years or less was 10% (95% Cl: 4 - 17%; I^2^= 73.33%; Q test = 14.67; P = 0.001) and in patients older than 60 years, it was 17% (95% Cl: 10 - 44%; I^2^= 55.40%; Q test = 22.53; P = 0.03) (Table 3). 

***The pooled prevalence of underlying diseases***

**Figure 4:**
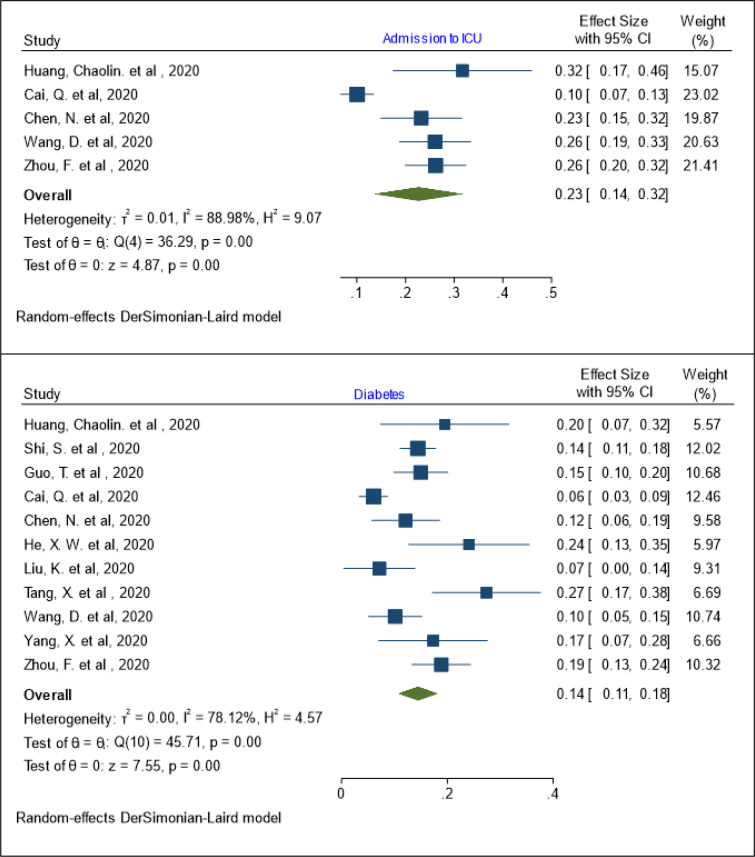
The pooled prevalence of admission to ICU and diabetes in patients with COVID-19

The pooled prevalence of admission to ICU has been estimated as 23% (95% Cl: 14 – 32 %; I^2^= 88.98%; Q test = 36.29; P = 0.001) (Figure 4). The results of meta regression showed that the prevalence of admission to ICU has no relationship with age (coefficient: 0.008, P: 0.164, 95% CI: -0.003, 0.021). The pooled prevalence of admission to ICU in patients aged 60 years or less was 23% (95% Cl: 12 - 34%; I^2^= 91.12%; Q test = 33.77; P = 0.001) (Table 3). The pooled prevalence of diabetes in patients with COVID-19 was 14% (95% Cl: 11 - 18%; I^2^= 78.12%; Q test = 45.71; P = 0.001) (Figure 4). The pooled prevalence of diabetes in patients aged 60 years or less was 13 % (95% Cl: 8 - 17%; I^2^= 77.47%; Q test = 26.63; P = 0.001) and in patients older than 60 years, it was 18 % (95% Cl: 12 - 24%; I^2^= 65.98%; Q test = 8.82; P = 0.03) (Table 3). The results of meta regression showed that the prevalence of admission to ICU has no relationship with age (coefficient: 0.053, P: 0.174, 95% CI: -0.023, 0.130).

**Figure 5:**
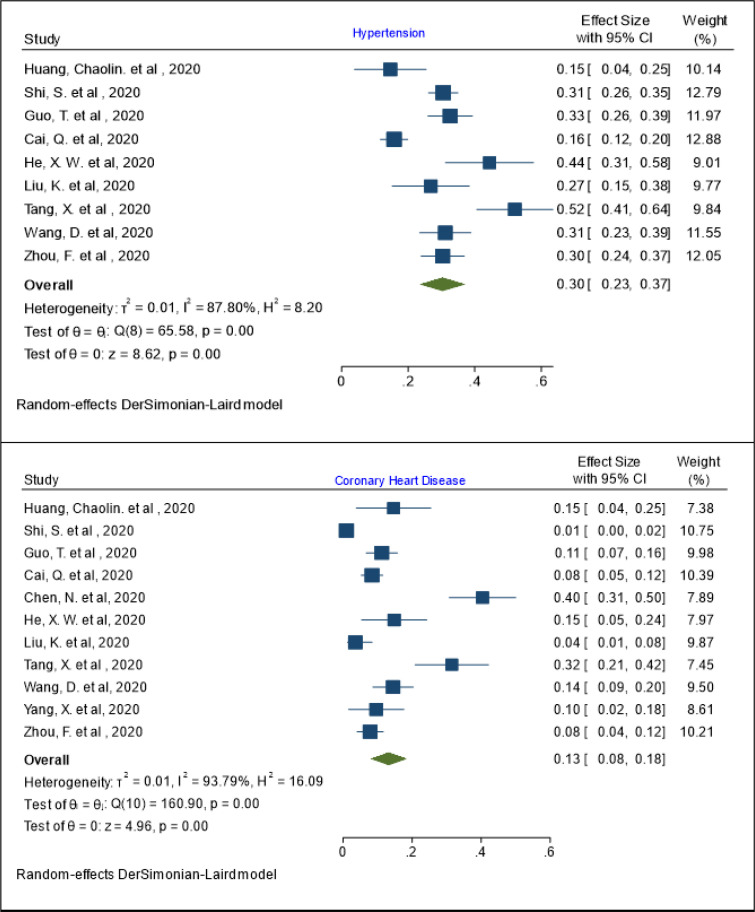
The pooled prevalence of hypertension and coronary heart disease in patients with COVID-19

**Figure 6:**
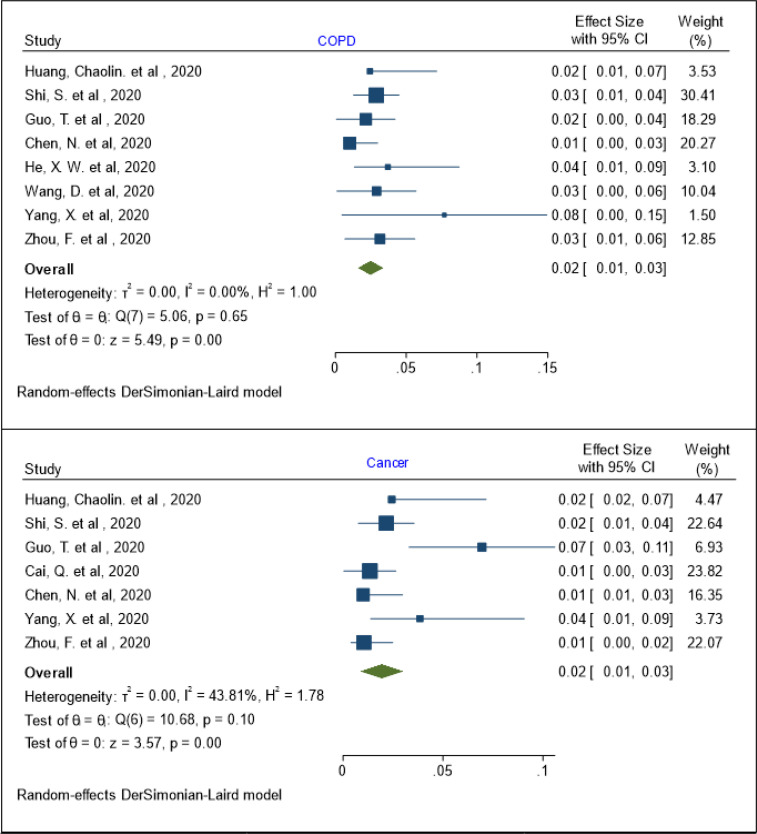
The pooled prevalence of chronic obstructive pulmonary disease (COPD) and cancer in patients with COVID-19

The prevalence of hypertension in patients with COVID-19 was 30% (95% Cl: 23 - 37%; I^2^= 87.80%; Q test = 65.58; P = 0.001) (Figure 5). The results of meta regression showed that the prevalence of hypertension has a relationship with age (coefficient: 0.157, P: 0.029, 95% CI: 0.016, 0.029). Also, the pooled prevalence rates of coronary heart disease and COPD were 13% (95% Cl: 8 - 18%; I^2^= 93.79%; Q test = 160.90; P = 0.001) and 2% (95% Cl: 1 - 3%; I^2^= 0.0%; Q test = 5.06; P = 0.65), respectively (Figure 5 and 6). 

The results of meta regression showed that the prevalence of coronary heart disease and COPD has no relationship with age (coefficient: 0.106, P: 0.092, 95% CI: -0.017, 0.230) and (coefficient: -0.005, P: 0.537, 95% CI: -0.023, 0.012), respectively. Also, the pooled prevalence of cancer in patients with COVID-19 was 2% (95% Cl: 1 - 3%; I^2^= 43.81%; Q test = 10.68; P = 0.10) (Figure 6). The prevalence of cancer has no relationship with age (coefficient: -0.006, P: 0.628, 95% CI: -0.031, 0.019). 

**Table 1:** The complex and simple search syntax in PubMed and Embase Databases

Databases	Complex and Simple Search Syntax
Embase	('covid 19'/exp OR 'coronavirus disease 2019'/exp) AND ('acute heart infarction'/exp OR 'acute cardiac infarction' OR 'acute heart infarction' OR 'acute myocardial infarction' OR 'heart infarction, acute' OR 'acute kidney failure'/exp OR 'acute kidney failure' OR 'acute kidney injury' OR 'acute kidney insufficiency' OR 'acute renal failure' OR 'acute renal insufficiency' OR 'kidney acute failure' OR 'kidney failure, acute' OR 'kidney insufficiency, acute' OR 'renal insufficiency, acute' OR 'liver injury'/exp OR 'acute liver injury' OR 'blunt liver trauma' OR 'hepatic damage' OR 'hepatic injury' OR 'hepatic lesion' OR 'hepatic trauma' OR 'injury, liver' OR 'liver damage' OR 'liver injury' OR 'liver lesion' OR 'liver parenchymal injury' OR 'liver trauma' OR 'liver wound' OR 'trauma, hepatic' OR 'trauma, liver' OR 'wound, liver' OR 'shock'/exp OR 'cardiovascular collapse' OR 'circulation shock' OR 'circulatory collapse' OR 'circulatory shock' OR 'incremental shock' OR 'nonseptic shock' OR 'shock' OR 'shock index' OR 'shock intensity' OR 'shock syndrome' OR 'shock, surgical' OR 'surgical shock' OR 'acute respiratory tract disease'/exp OR 'acute respiratory disease' OR 'acute respiratory tract disease' OR 'respiratory disease, acute' OR 'adult respiratory distress syndrome'/exp OR 'ards' OR 'acute respiratory distress syndrome' OR 'adult respiratory distress' OR 'adult respiratory distress syndrome' OR 'lung shock' OR 'posttraumatic lung failure' OR 'posttraumatic pulmonary insufficiency' OR 'respiratory distress syndrome, acute' OR 'respiratory distress syndrome, adult' OR 'respiratory distress, adult' OR 'shock lung')
PubMed	(((((Acute[All Fields] AND ("heart"[MeSH Terms] OR "heart"[All Fields] OR "cardiac"[All Fields]) AND ("wounds and injuries"[MeSH Terms] OR ("wounds"[All Fields] AND "injuries"[All Fields]) OR "wounds and injuries"[All Fields] OR "injury"[All Fields])) OR ("acute kidney injury"[MeSH Terms] OR ("acute"[All Fields] AND "kidney"[All Fields] AND "injury"[All Fields]) OR "acute kidney injury"[All Fields])) OR (Acute[All Fields] AND Respiratory[All Fields] AND ("wounds and injuries"[MeSH Terms] OR ("wounds"[All Fields] AND "injuries"[All Fields]) OR "wounds and injuries"[All Fields] OR "injury"[All Fields]))) OR (Acute[All Fields] AND ("myocardial infarction"[MeSH Terms] OR ("myocardial"[All Fields] AND "infarction"[All Fields]) OR "myocardial infarction"[All Fields]))) OR ("respiratory distress syndrome, adult"[MeSH Terms] OR ("respiratory"[All Fields] AND "distress"[All Fields] AND "syndrome"[All Fields] AND "adult"[All Fields]) OR "adult respiratory distress syndrome"[All Fields] OR ("acute"[All Fields] AND "respiratory"[All Fields] AND "distress"[All Fields] AND "syndrome"[All Fields]) OR "acute respiratory distress syndrome"[All Fields])) AND (("COVID-19"[All Fields] OR "COVID-2019"[All Fields] OR "severe acute respiratory syndrome coronavirus 2"[Supplementary Concept] OR "severe acute respiratory syndrome coronavirus 2"[All Fields] OR "2019-nCoV"[All Fields] OR "SARS-CoV-2"[All Fields] OR "2019nCoV"[All Fields] OR (("Wuhan"[All Fields] AND ("coronavirus"[MeSH Terms] OR "coronavirus"[All Fields])) AND (2019/12[PDAT] OR 2020[PDAT]))) OR (("coronavirus"[MeSH Terms] OR "coronavirus"[All Fields]) AND 2019[All Fields]))

**Table 2:** Patient characteristics, underlying diseases, and comorbid conditions in patients with COVID-19

Authors (Years) (Country)	Study population (Type of Studies)	Sample size (Male , Female)	Age (Year)	Chronic Medical Illness	Comorbid Conditions
Huang, Chaolin. et al. (2020) (28) China	Patients with 2019-nCoV pneumonia (Cross sectional)	41 (30 M, 11 F)	49	ICU Care (5/13: 38%) No ICU Care (1/28: 4 %) ICU Admitted (13/41: 31.7%) Diabetes (8/41: 20 %) Hypertension (6/41: 15 %) Cardiovascular disease (6/41: 15%) COPD (1/41: 2%) Cancer (1/41: 1%)	Acute respiratory distress Syndrome (12/41: 29%) RNAaemia (6/41: 15%) Acute cardiac injury (5/41: 12%) Acute kidney injury (3/41: 7%) Shock (3/41: 7%) Secondary infection (4 /41: 10 %)
Shi, S. et al. (2020) (18) China	Patients with 2019-nCoV (Cohort)	416 (205 M, 211 F)	64 (21 – 95)	With Cardiac Injury (42/82: 51.2%) Without Cardiac Injury (15/334: 51.2%) Hypertension (127/416:30.5%) Diabetes (60/416: 14.4%) Coronary heart disease (4/416: 10.6%) Cancer (9/416: 2.2%) COPD (12/416: 2.9%)	HBV (4 / 416: 1%) Acute cardiac injury (82/416: 19.7%) ARDS (97/416: 23.3%) Acute kidney injury (8/416: 1.9%) Anemia (13/416: 3.1%) Coagulation disorder (12/416: 2.9%)
Guo, T. et al. (2020) (19) China	Patients with 2019-nCoV (Cohort)	187 (91 M, 96 F)	58	Elevated TnT Level (31/52: 4.5 %) Normal TnT Level (12/135: 8.9 %) Hypertension (61/187:32.6%) Diabetes (28/187: 15%) Coronary heart disease (21/187: 11.2%) Cancer (13/187: 7%) COPD (4/187: 2.1%)	ARDS (46/187: 24.6%) Acute kidney injury (18/187: 14.6%) Acute liver injury (19/187: 15.4%) Coagulation disorder (42/187: 34.1%)
Cai, Q. et al. (2020) (20) China	Patients with 2019-nCoV (Cross sectional)	298 (145 M, 153 F)	47.5	Length of hospital stay (20.5 [15-26] days) Need for ICU (30/298: 10.1%) Cardiovascular Disease (25/298: 8.39%) Hypertension (47/298: 15.8%) Diabetes (18/298: 6.04%) Cancer (4/298: 1.3%)	Acute liver injury (44/298: 14.8%) Acute kidney injury (17/298: 5.7%) Acute cardiac injury (20/298: 6.7%)
Chen, N. et al. (2020) (21) China	Patients with 2019-nCoV (Cross sectional)	99 (67 M, 32 F)	20 - 90	Cardiovascular and cerebrovascular diseases (40/99: 40 %) Diabetes (12/99: 12%) Respiratory system disease (1/99: 1%) Cancer (1/99: 1 %) ICU Admitted (23/99: 23%)	ARDS (17/99: 17%) Acute respiratory injury (8/99: 8 %) Shock (4/99: 4%)
He, X. W. et al. (2020) (22) China	Patients with 2019-nCoV (Cross sectional)	54 (-)	68 (59.8 -74.3)	Hypertension (24/54: 44.4%) Diabetes (13/54: 24.1%) Coronary Heart Disease (8/54: 14.8%) COPD (2/54: 3.7%)	Acute cardiac injury (24/54 :44.4%)
Lian, J. et al. (2020) (23) China	Patients with 2019-nCoV (Retrospective)	788 (407 M, 381 F)	All age	Admitted ICU (27/788: 3.42%) Hypertension (126/788: 16%) Coronary Heart Disease (11/788: 1.4%) Diabetes (57/788: 7.23 %) Liver disease (31/788: 3.93%) COPD (3/788: 0.38%) Cancer (6/788: 0.76%)	Acute respiratory distress (58/788: 7.36%) Shock (2/788: 0.25%) Liver function abnormality (82/788: 10.4%) Acute kidney injury (13/788: 1.64%)
Liu, K. et al. (2020) (24) China	Patients with 2019-nCoV (Retrospective)	56 (31 M, 25 F)	47-68	Hypertension (15/56: 26.78%) Coronary Heart Disease (2/56: 3.57%) Diabetes (4/56: 7.14 %) Liver disease (1/56: 1.78%)	Acute respiratory distress syndrome (6/56: 10.71%) Acute heart injury (7/56: 12.5%) Acute liver and kidney injury (10/56: 17.85%) Shock (3/56: 5.35%)
Tang, X. et al. (2020) (25) China	Patients with 2019-nCoV (Retrospective)	73 (45 M, 28 F)	67	Hypertension (38/73: 52.1%) Coronary Heart Disease (23/73: 31.5%) Diabetes (20/73: 27.4%) Kidney disease (3/73: 4.1%)	Leukocytopenia (60/73: 82.2%) Shock (23/73: 31.5%) Acute kidney injury (13/73: 17.8%) Liver dysfunction (33/73: 54.2%)
Wang, D. et al. (2020) (26) China	Patients with 2019-nCoV (Retrospective)	138 (75 M, 63 F)	56	ICU (36/138: 26.08) Hypertension (43/138: 31.2%) Cardiovascular disease (20/138: 14.5%) Diabetes (14/138: 10.1%) Cancer (10/138: 7.2 %) COPD (4/138: 2.9%) HIV (2 /138: 1.4%)	Shock (12/138: 8.7 %) Acute cardiac injury (10/138: 7.2%) Arrhythmia (23/138: 16.7%) ARDS (27/138: 19.6%) AKI (5/138: 3.6%) kidney disease (4/138: 2.9%) Liver disease (4/138: 2.9%)
Yang, X. et al. (2020) (28) China	Patients with 2019-nCoV (Retrospective)	52 (35 M, 17 F)	59.7	Cardiovascular disease (5/52: 10%) Diabetes (9/52: 17%) Cancer (2/52: 4 %) COPD (4/52: 8%) Dementia (1/52: 2%)	Acute respiratory distress (35/52: 67%) Acute kidney injury (15/52: 29%) Liver dysfunction (15/52: 29%) Acute cardiac injury (12/52: 23%)
Zhou, F. et al (2020) (27) China	Patients with 2019-nCoV (Retrospective)	191 (119 M, 72 F)	56	Hypertension (26/54: 48%) Cardiovascular disease (13/54: 24%) Diabetes (17/54: 31%) COPD (4/54: 7%) Chronic kidney disease (2/54: 2%) ICU Admission (39/54: 72%)	ARDS (50/54: 93%) Shock (38/54: 70%) Heart failure (28/54: 52%) Acute kidney injury (27/54: 50%) Acute respiratory distress (53/54: 98%)

ICU: intensive care unit; COPD: chronic obstructive pulmonary disease; HBV: hepatitis B virus; ARDS: acute respiratory distress syndrome; TnT: Troponin T; HIV: human immunodeficiency virus; AKI: acute kidney injury.

**Table 3:** Summary of prevalence estimates [95 % confidence intervals (CIs)] for underlying diseases and comorbid conditions in patients with COVID-19 based on their age group according to the included studies

Subgroup	Study (n)	Pooled Prevalence	Between studies			Between subgroups	
			I2 (%)	P*	Q	Q	P*
Acute Respiratory Injury > 60 Years ≤ 60 Years	3 2	43 % (6 – 80 %) 19 % (1– 37 %)	80.37 98.58	0.02 0.00	5.09 140.77	1.26	0.26
Acute Kidney Injury > 60 Years ≤ 60 Years	6 3	11 % (5 – 16 %) 9 % (3– 15 %)	59.32 62.35	0.24 0.07	5.09 5.77	0.17	0.68
Acute Liver Injury > 60 Years ≤ 60 Years	4 2	20 % (7 – 34 %) 15 % (11– 19 %)	55.40 0.0	0.09 0.58	4.99 0.31	0.55	0.46
Acute Respiratory Distress Syndrome > 60 Years ≤ 60 Years	2 3	21 % (15 – 27 %) 25 % (19– 31 %)	50.58 64.97	0.15 0.08	2.02 5.71	0.82	0.36
Shock > 60 Years ≤ 60 Years	2 4	17 % (10 – 44 %) 10 % (4– 17 %)	69.55 73.33	0.03 0.001	22.53 14.67	0.24	0.63
Admission to ICU > 60 Years ≤ 60 Years	1 4	23 % (15 – 32 %) 23 % (12– 34 %)	- 91.12	- 0.001	- 33.77	0.01	0.94
Diabetes > 60 Years ≤ 60 Years	4 7	18 % (12 – 24 %) 13 % (8– 17 %)	65.98 77.47	0.03 0.001	8.82 26.63	1.84	0.18
COPD > 60 Years ≤ 60 Years	3 5	2 % (1 – 4 %) 3 % (1– 4 %)	17.98 0.0	0.30 0.69	2.44 2.24	0.35	0.56
Cancer > 60 Years ≤ 60 Years	2 5	2 % (1 – 4 %) 3 % (1– 3 %)	0.0 59.14	0.35 0.04	0.88 9.79	0.37	0.54
CHD > 60 Years ≤ 60 Years	4 7	22 % (1 – 42 %) 9 % (7– 12 %)	97.04 43.48	0.001 0.10	101.38 10.62	1.37	0.24

* Heterogeneity. ICU: intensive care unit; COPD: chronic obstructive pulmonary disease; CHD: coronary heart disease.

## Discussion

The results of this study showed that the pooled prevalence of acute respiratory injury in patients with COVID-19 has been estimated as 34% (95% Cl: 10 – 57%). Also, the prevalence rates of acute kidney injury, acute liver injury, acute respiratory distress syndrome, and shock have been estimated as 10% (95% Cl: 6 - 14%), 19% (95% Cl: 10 - 27%), 23% (95% Cl: 19 - 27%), and 12% (95% Cl: 5 – 19 %), respectively. COVID-19 is a respiratory infectious disease that causes the most damage to the lungs. People with the disease suffer from shortness of breath and severe cough. The virus infects and kills lung ciliated cells, which are responsible for clearing viruses. When they are destroyed, the airways become filled with waste and fluids, thus activating the person’s immune system, which sends immune cells to the lungs to destroy the virus. In this process, however, the healthy tissues are also damaged and the lungs become inflamed. This inflammation affects the oxygen supply capacity of the lungs and can lead to death in acute cases. The US Center for Disease Control and Prevention reported that COVID-19 was a threat to public health, and that older people with chronic medical conditions such as diabetes were at higher risk for severe illness and experiencing the side effects. Studies have shown that the risk of developing severe side effects of COVID-19 in people with diabetes is equal to normal people when the diabetes is controlled. On the other hand, the results of a study by Leung, Janice M. et al. showed that active smoking and COPD increased the expression of ACE-2 gene in the lower airways, which may to some extent justify the increased risk of COVID-19 in these populations (30). 

The results of this study showed that the prevalence of hypertension in COVID-19 patients (I^2^ index) was 91.45% with a confidence interval of 29.0 (95%: CI: 0.22 - 0.35). It is estimated that increased age has a positive effect on this rate. Based on the results of recent studies, hypertension, cardiovascular disease, diabetes, kidney disease, smoking and COPD were among the most important underlying diseases among COVID-19 patients (31, 32). COVID-19 is transmitted via the respiratory system. The disease mainly causes (severe) respiratory infections. All people are susceptible to the virus, but older people and those with underlying diseases are more likely to be infected and exposed to side effects. Current findings have shown that mortality is very high in people with underlying diseases.  In a study titled “Evaluation of Clinical Symptoms in People with COVID-19”, Zhang J-j et al. showed that cardiovascular disease is the most prevalent underlying disease among COVID-19 patients according to existing medical evidence. It is worth noting that this pattern has also been found in Middle East Respiratory Syndrome (MERS) (31). Based on the results, hypertension is one of the most common comorbid diseases, which has a direct correlation with age in patients with coronavirus.

In the study of diabetes in patients with coronavirus, the results of this study showed that the prevalence of diabetes (I^2^ index) was 81.52% with a confidence interval of 13.0 (95%: CI: 0.10 - 0.17). It is estimated that increased age has a positive effect on this rate. Xiaobo Yang et al. concluded that 22% of patients with coronavirus have diabetes. In another study, among 1,099 patients with a definitive diagnosis of coronavirus, 16.2% had an active type of diabetes. Research has shown that diabetes increases the risk of developing diseases such as influenza and pneumonia by reducing the power of the immune system, while controlling the rate of hyperglycemia reduces the risks. In fact, diabetes has been introdused as a risk factor for pandemic diseases such as influenza, COVID-19 and severe respiratory failure. On the other hand, information on the prevalence of COVID-19 among diabetic patients is currently limited; 42.3% of COVID-19-related death cases reported in Wuhan, China had diabetes. Another study on 150 patients with 68 deaths and 82 recovered patients in Wuhan found that presence of underlying diseases was an important predictor of mortality. According to the results, diabetes is one of the most common underlying diseases, which is directly related to age in patients with coronavirus (33).

In the study of heart failure in patients with coronavirus, the results showed that the prevalence of diabetes (I^2^ index) was 93.54% with a confidence interval of 0.11 (95%: CI: 0.08 - 0.14). It is estimated that increased age has a positive effect on this prevalence. Studies show that coronavirus can increase the risk of heart failure and myocarditis, while increasing the patient’s resistance to treatment and increasing the risk of death from heart failure. Reports in Wuhan, China, have shown that heart failure is observed in 5 out of every 41 COVID-19 patients with increased sensitivity to heart markers such as troponin. Patients with palpitations and chest tightness with respiratory symptoms, such as fever and cough, were later diagnosed with COVID-19. On the other hand, among the casualties of COVID-19, 11.8% had high troponin levels without heart symptoms. Therefore, it is seen in COVID-19 patients due to systemic inflammatory response and immune system disorders during disease progression. A 12-year follow-up of 25 patients with various types of coronaviruses showed that 68% had hyperlipidemia and 44% had heart failure. According to the results of studies, heart failure is one of the most common associated diseases, which directly correlates with age in patients with coronavirus (34, 35). In the study of cancer in patients with coronavirus, it was shown that the prevalence of cancer (I^2^ index) was 52.54% with a confidence interval of 0.02. It is believed that increase in age has a positive effect on this prevalence. A study on 1590 COVID-19 patients in Wuhan, China, found that 18 patients had cancer, among whom only 4 underwent surgery or chemotherapy in the previous month, and 12 had recovered from cancer and had no clear indication of weakened immune system. Therefore, it can be argued that patients with cancer will be prone to all kinds of infections due to receiving immunosuppressive drugs, so these patients are also more prone to coronavirus and have weaker diagnostic markers. As a result, chemotherapy can be delayed to reduce the mortality rate of these people during the coronavirus outbreak; also, stronger personal protection regulations, and closely monitoring the treatment of these people, especially the older patients, may help reduce their risk of infection. According to the results, cancer is one of the most common underlying diseases, which is directly related to age in patients with coronavirus (36, 37). 

## Limitation:

One of the limitations of our study is the high heterogeneity in some categories. Also, in the results of included studies, potential confounder factors were not reported. So, subgroup analysis was done based on age, alone. In addition, the screening of articles found via the initial search, data extraction, and quality assessment of included articles may have been influenced by personal judgments.

## Conclusion

In summary, the results of the present study showed that in patients with SARS-CoV-2 infection, hypertension, cardiovascular disease, smoking, and diabetes were the most common underlying disorders. Therefore, due to the long and asymptomatic incubation period, it is often recommended that people with chronic diseases follow health advice more closely and avoid contact with other people. Also, comorbid conditions like hypertension, acute liver injury, acute kidney injury, acute respiratory distress syndrome, shock, diabetes, and coronary heart disease seem to be a predisposing factor for symptomatic and severe COVID-19 infection. 

## Ethical approval and consent to participate

Not applicable.

## Consent for Publication

Not applicable.

## Competing Interests

The authors declare that they have no competing interests.

## Funding

This research did not receive any specific grant from funding agencies in the public, commercial, or not-for-profit sectors.

## Authors' Contributions

SKH, and YM conceptualized the idea for this review, formulated the review question, and objectives, assisted with the development of the final search strategy, contributed to the data analysis/ interpretation, and writing the manuscript. RKH, HM, SKH and YM contributed to the conceptualization of the final review question, formulation of the review objectives, data analysis/interpretation, and writing the manuscript. All authors equally contributed to the formulation of the review question/objectives, development of the search strategy, conducting the searches, data extraction, data analysis/interpretation, and writing the manuscript. All authors read and approved the final manuscript.

## Data Availability

Input data for the analyses are available by the corresponding author on request.
